# Equilin in conjugated equine estrogen increases monocyte-endothelial adhesion via NF-κB signaling

**DOI:** 10.1371/journal.pone.0211462

**Published:** 2019-01-30

**Authors:** Fumitake Ito, Taisuke Mori, Yosuke Tarumi, Hiroyuki Okimura, Hisashi Kataoka, Yukiko Tanaka, Akemi Koshiba, Jo Kitawaki

**Affiliations:** Department of Obstetrics and Gynecology, Kyoto Prefectural University of Medicine, Graduate School of Medical Science, Kyoto, Japan; Universita degli Studi di Padova, ITALY

## Abstract

The adhesion of monocytes to endothelial cells, which is mediated by adhesion molecules, plays a crucial role in the onset of atherosclerosis. Conjugated equine estrogen, which is widely used for estrogen-replacement therapy, contains both estrone sulfate and various nonhuman estrogens, including equilin. To investigate the association between various estrogen types and atherosclerosis risk, we examined their effect on adhesion-molecule expression in human umbilical vein endothelial cells (HUVECs). In estrogen-treated HUVECs, the mRNA and protein expression levels of adhesion molecules were quantified by real-time polymerase chain reaction and enzyme immunoassay. Additionally, a flow-chamber system was used to assess the effects of estrogens on the adherence of U937 monocytoid cells to HUVECs. Equilin, but not 17β-estradiol (E2) or other types of estrogen, significantly increased the mRNA (*P* < 0.01) and protein (*P* < 0.05) expression of the adhesion molecules E-selectin and intercellular adhesion molecule-1 as compared with levels in controls. Equilin treatment increased the adherence of U937 monocytoid cells to HUVECs relative to the that in the control (*P* < 0.05), decreased estrogen receptor (ER)β expression, and increased the expression of proteins involved in nuclear factor kappa-B (NF-κB) activation relative to levels in controls. Furthermore, the accumulation of NF-κB subunit p65 in HUVEC nuclei was promoted by equilin treatment. By contrast, E2 treatment neither increased the number of adhered monocytoid cells to HUVECs nor altered the expression of ERβ or NF-κB-activating proteins. Our findings suggest that in terms of the adhesion of monocytes at the onset of atherosclerosis, E2 may be preferable for estrogen-replacement therapy. Further studies comparing equilin treatment with that of E2 are needed to investigate their differential impacts on atherosclerosis.

## Introduction

Hormone-replacement therapy (HRT) is commonly prescribed for postmenopausal women to treat climacteric disorders caused by estrogen deficiency and to reduce the risk of osteoporosis. Prior to 2002, HRT was believed to have additional benefits in preventing cardiovascular events based on observational studies, which suggested that HRT approximately halves the risk of cardiovascular disease in postmenopausal women [1]. However, in 2002, a large-scale, randomized trial by the Women’s Health Initiative (WHI) showed that HRT offers no cardiovascular benefits, and from that point forward, HRT has continued to be the subject of much discussion and speculation [2]. The North American Menopause Society states that most observational studies support the potential benefits of systemic HRT in reducing coronary heart disease (CHD), whereas most randomized controlled trials do not [3].

One explanation for the conflicting results among clinical trials may involve the fact that diverse clinical studies were conducted with the use of different HRT types and regimens [4]. We previously reported the adverse effects of medroxyprogesterone acetate, which is co-administered in most HRT regimens, on endothelial cells and its association with the risk of atherosclerosis development [5]; however, few studies have compared the effects of different estrogen types on cardiovascular events associated with estrogen-replacement therapy (ERT) [6,7]. Furthermore, few basic studies have investigated the cardiovascular benefits associated with various types of estrogen [8].

Conjugated equine estrogen (CEE), a type of estrogen that is commonly administered in most ERT regimens, is derived from the urine of pregnant horses. CEE consists of a mixture of estrogens, such as equilin (Eq) or equilenin (EL) [9]. As CEE consists of non-human components, when evaluating the effect of CEE on CHD risk, each component of CEE requires investigation. However, in most clinical trials, CEE is not distinguished from other estrogens, such as 17β-estradiol (E2). There is accumulating evidence that E2 exerts beneficial effects on the endothelium in terms of atherosclerosis development, including reducing low-density lipoprotein cholesterol, facilitating nitric oxide-mediated vasodilation, and inhibiting the response of blood vessels to injury [10–12]; however, the effect of each component of CEE on endothelial cells remains unknown.

The adhesion of circulating monocytes to endothelial cells constitutes an important initial event in early atherogenesis, with intercellular adhesion requiring specific receptor-ligand interactions between endothelial cell-adhesion molecules and adhesion receptors on monocytes [12–14]. We previously reported that various progestogens modify the expression of adhesion molecules on human umbilical vein endothelial cells (HUVECs) *in vitro* [5,15,16]; however, the association between various estrogens and the expression of adhesion molecules remains elusive.

In this study, to investigate whether various types of estrogen are associated with atherosclerosis risk, we examined the effects of various estrogens on the expression of cell-adhesion molecules and the actual adherence of monocytoid cells to HUVECs under flow conditions. Additionally, we explored the mechanisms underlying changes in adhesion-molecule expression during estrogen treatment.

## Materials and methods

### Estrogens

E2 and estrone (E1) were purchased from Nacalai Tesque, Inc. (Kyoto, Japan). Estriol (E3), Eq, and ethinylestradiol (EE) were purchased from Sigma-Aldrich (St. Louis, MO, USA). Estetrol (E4) was purchased from Toronto Research Chemicals, Inc. (North York, Ontario, Canada). EL was purchased from Alfa Chemistry (Stony Brook, NY, USA).

### Cell cultures and treatments

This protocol has been approved by the Kyoto Prefectural University of Medicine Institutional Review Board. Female infant umbilical cords were obtained from women who had given birth by normal delivery without complications. HUVECs were separated using the method described in our previous report [5]. Briefly, HUVECs were cultured at 37°C under a humidified atmosphere of 5% CO_2_ in 75-cm^2^ tissue-culture flasks until cells reached pre-confluence. Cell purity was confirmed by the appearance of a typical cobblestone morphology and by the presence of von Willebrand-factor antigen. The medium was changed to minimum essential medium (without phenol red) containing 4% charcoal-treated fetal bovine serum, penicillin (100 IU/mL), and streptomycin (100 μg/mL), and the cells were pre-cultured for 12 hours. Then, the cells were incubated with incubation medium containing estrogens for 24 hours.

### Real-time polymerase chain reaction (PCR) analysis

Total RNA was extracted from HUVECs using the RNeasy Mini Kit (Qiagen, Venlo, Netherlands) according to the manufacturer’s instructions. RNA (1 μg) from each sample was reverse-transcribed to cDNA and amplified using ReverTra Ace qPCR RT master mix (Toyobo, Osaka, Japan). Gene expression levels were analyzed using real-time PCR. The forward and reverse primers for E-selectin, P-selectin, intercellular adhesion molecule-1 (ICAM-1), and vascular adhesion molecule-1 (VCAM-1) are listed in S1 Table. The cycle threshold (Ct) values obtained were used to quantify the relative expression of the genes of interest. The Ct values were first normalized to that of the internal control gene (*GAPDH*), and then the fold changes of target genes (ΔΔCt) in all groups were calculated and represented as relative expression values.

### Enzyme immunoassay (EIA)

The protein expression of adhesion molecules was examined by EIA as described previously [5]. Briefly, HUVEC monolayers in 96-well plates were fixed by the addition of 4% paraformaldehyde. To create a standard curve, each 96-well plate was coated with diluted capture antibody (R&D Systems, Minneapolis, MN, USA), and standards were added according to the manufacturer’s instructions. Mouse anti-E-selectin monoclonal, mouse anti-P-selectin monoclonal, mouse anti-ICAM-1 monoclonal, and mouse anti-VCAM-1 monoclonal antibodies (1 μg/mL; R&D Systems) were added to each plate. The wells were washed twice with wash buffer, and biotinylated goat anti-mouse antibody (Dako, Carpinteria, CA, USA) was added. The wells were again washed twice with wash buffer, followed by the addition of streptavidin-horseradish peroxidase complex (Dako). After two washes with wash buffer, *O*-phenylenediamine dihydrochloride in citric acid phosphate buffer (pH 5.0) was added and reacted for 5 minutes at room temperature. The reaction was stopped by the addition of 1 mol/L sulfuric acid. Absorbance was measured at 490 nm using an enzyme-linked immunosorbent assay reader (Model 550; Bio-Rad, Hercules, CA, USA).

### Immunofluorescence analysis

After HUVECs grown on glass slides were treated with 4% paraformaldehyde, the slides were incubated first with 0.1 mol/L glycine in phosphate-buffered saline (PBS) and then with 0.1% Triton X-100 in PBS. The slides were washed and then incubated with 5% swine serum in PBS for 1 hour.

For nuclear factor kappa-B (NF-κB) subunit p65 immunostaining, slides were incubated with diluted mouse anti-NF-κB p65 antibody (Santa Cruz Biotechnology, Dallas, TX, USA). After washing with PBS three times, the slides were incubated with Alexa Fluor 488 goat anti-mouse IgG antibody (Cell Signaling Technology, Danvers, MA, USA) for 1 hour. Then, the slides were washed and mounted in Vectashield mounting medium with 4′,6-diamidino-2-phenylindole (Vector Laboratories, Burlingame, CA, USA). Images were captured with a fluorescence microscope.

### Flow-chamber system

We assessed the effect of shear stress on the adherence of U937 monocytoid cells to HUVEC monolayers. To produce well-defined shear stress, we used a flow-chamber system previously described by Gerszten et al. [17], with modifications as previously described [18]. Briefly, the chamber consisted of a glass slide with a confluent HUVEC monolayer that was attached to a polycarbonate base. Two flat monolayer surfaces were held approximately 270 μm apart by a silastic rubber gasket (Dow Corning, Midland, MI, USA). Flow across the monolayers was controlled with a syringe pump (Harvard Apparatus, South Natick, MA, USA), and a U937 monocytoid cell suspension (10,000 cells/mL) in Hank’s balanced salt solution was perfused through the flow chamber. Experiments were videotaped using a color camera mounted on an inverted microscope, and the number of adherent U937 monocytoid cells was counted after perfusion of cells for 5 minutes.

### Western blotting analysis

Cellular protein extracts were collected using radioimmunoprecipitation buffer (Nacalai Tesque) and then mixed with sodium dodecyl sulfate (SDS) sample buffer [62.5 mmol/L Tris-HCl (pH 6.8), 10% glycerol, 1% SDS, 0.1% 2-mercaptoethanol, and 1 mmol/L phenylmethylsulfonyl fluoride] and heated for 5 minutes at 90°C. The lysates were loaded onto polyacrylamide gels, subjected to electrophoresis, and transferred to a polyvinylidene difluoride membrane. The blot was incubated with a blocking buffer (5% skim milk, Tris-buffered saline, Tween-20) for 30 minutes at room temperature, then incubated with the appropriate primary antibody in blocking buffer overnight at 4°C. The blot was incubated with the appropriate secondary antibody in blocking buffer for 1 hour at room temperature, and the signal was detected with Chemi-Lumi One Super (Nacalai Tesque).

The following antibodies were used at the indicated dilutions and were obtained from the indicated sources: rabbit anti-estrogen receptor (ER)β (1:500; Thermo Fisher Scientific, Waltham, MA, USA), rabbit anti-hypoxia-inducible factor (HIF)-1α (1:200; Novus Biologicals, Littleton, CO, USA), rabbit anti-inhibitor of κB (IκB)α (1:500; Cell Signaling Technology), rabbit anti-phosphorylated-IκBα (Ser32) (1:500; Cell Signaling Technology), and goat anti-IκB kinase (IKK)β (1:500; Santa Cruz Biotechnology).

### Small-interfering RNA (siRNA) transfection

For the siRNA experiment, HUVECs at >85% confluence were transiently transfected with scrambled or validated siRNA for p65 (Ambion; Thermo Fisher Scientific) using Lipofectamine LTX (Invitrogen, Carlsbad, CA, USA) according to the manufacturer’s instructions. RNA was extracted 48 hours after transfection, and cDNA was synthesized according to the methods described above. The suppression of p65 was confirmed by real-time PCR.

### Statistical analysis

All data are expressed as the mean ± standard error of the mean (SEM) of three separate experiments. Differences in estrogen stimulation were analyzed with one-factor analysis of variance (ANOVA), followed by the Bonferroni-Dunn test for multiple comparisons. Differences in siRNA experiments and suppression experiments were analyzed with two-way ANOVA and the post-hoc Tukey test. A *P* < 0.05 was considered significant.

## Results

### Equilin, but not other estrogens, increases mRNA expression of adhesion molecules in HUVECs

**Fig 1:**
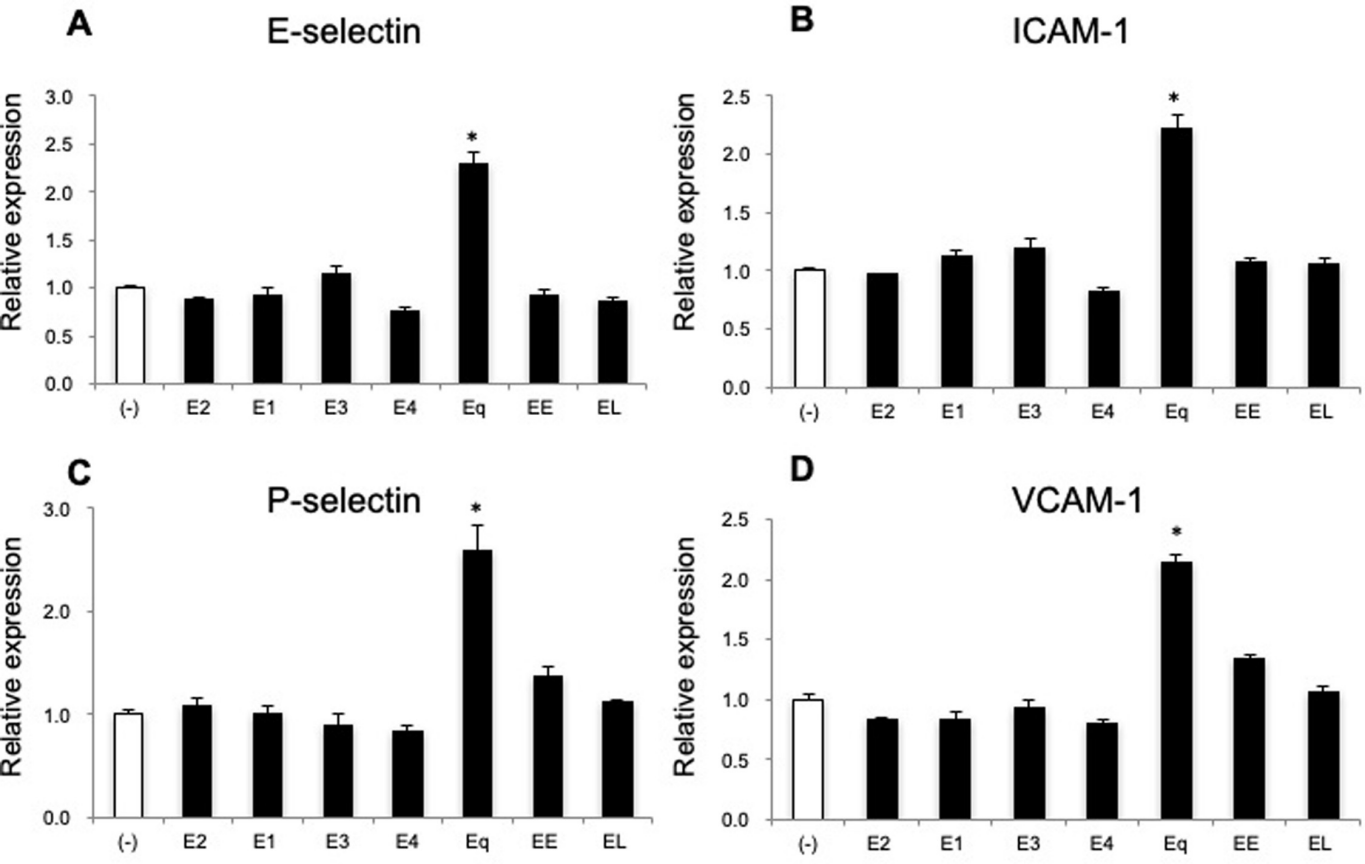
Eq upregulates mRNA expression of adhesion molecules in HUVECs. Steroid-deprived and serum-starved HUVECs were treated for 24 hours with various estrogens (1 nmol/L E2, 1 nmol/L E1, 1 nmol/L E3, 1 nmol/L E4, 1 nmol/L Eq, 1 nmol/L EL, or 100 pmol/L EE). The relative mRNA expression of genes encoding (**A**) E-selectin, (**B**) ICAM-1, (**C**) P-selectin, and (**D**) VCAM-1 was measured by real-time PCR. Data are expressed as the mean ± SEM of three experiments involving assays performed in triplicate. *P < 0.05 vs. vehicle alone. E2; 17β-estradiol, E1; estrone, E3; estriole, E4; estetrol, Eq; equilin, EL; equilenin, EE; ethinylestradiol.

First, we examined how estrogens affect cell-adhesion molecules in HUVECs by quantitative real-time PCR. Our preliminary study revealed that Eq treatment in the range of 100 pmol/L to 10 nmol/L resulted in the dose-dependent upregulation of adhesion-molecule expression. In addition, Eq treatment for 6 to 24 hours upregulated adhesion-molecule expression in a time-dependent manner (S1 Fig). Considering clinical use and physiological activities, we fixed estrogen concentrations at 1 nmol/L, with the exception of EE, which was used at 100 pmol/L. The results revealed that the mRNA levels of adhesion molecules in HUVECs treated with Eq were ~2.6-fold higher than the levels observed in the control (*P* < 0.05; Fig 1A–1D). By contrast, there were no significant differences in the expression of cell-adhesion molecules between HUVECs treated with other estrogens and the control.

### Equilin, but not other estrogens, increases protein levels of adhesion molecules in HUVECs

**Fig 2:**
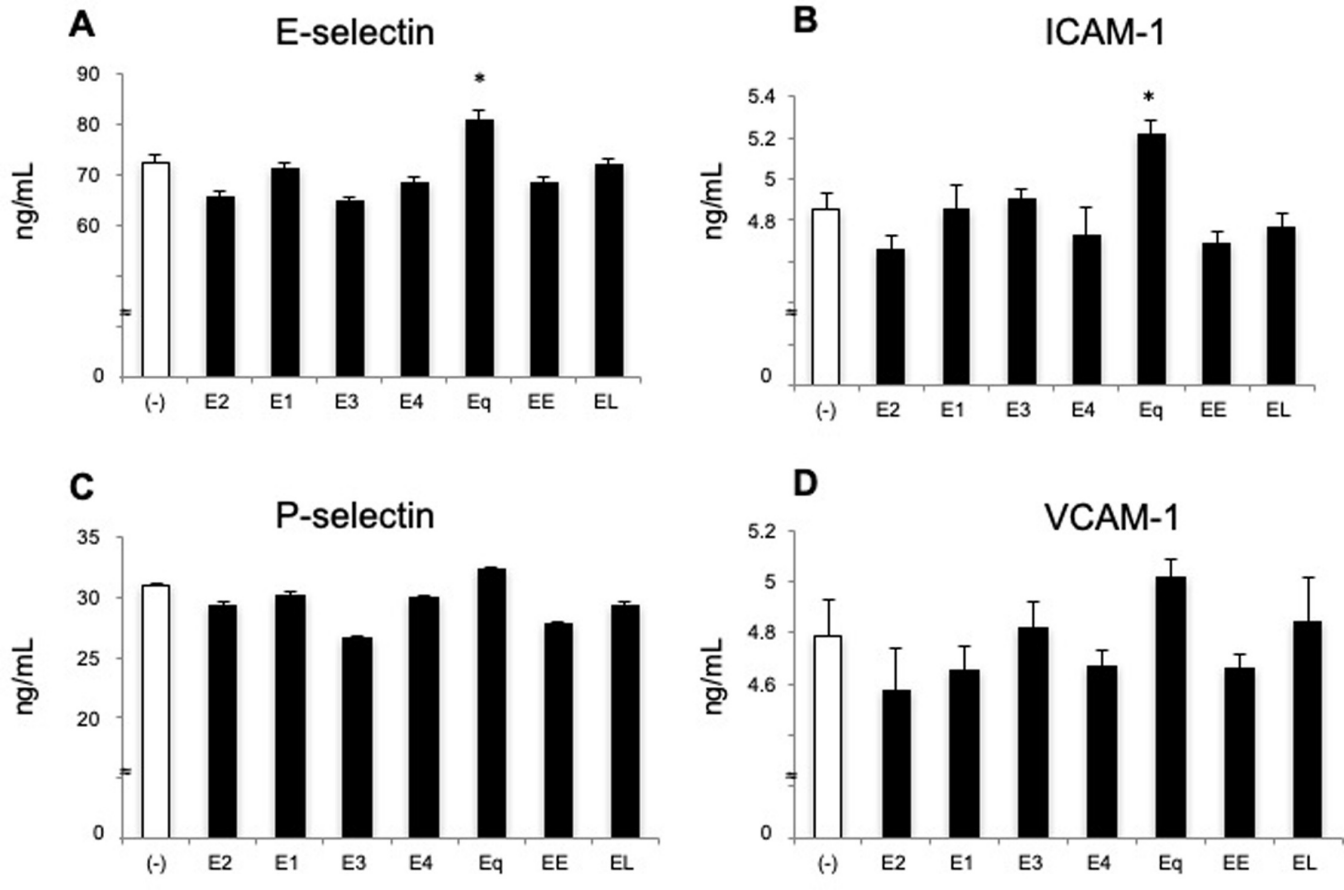
Eq upregulates protein expression of adhesion molecules in HUVECs. HUVECs were treated with various estrogens as described in Fig 1. (**A**) E-selectin, (**B**) ICAM-1, (**C**) P-selectin, and (**D**) VCAM-1 protein levels were quantified by EIA. Data are expressed as the mean ± SEM of four experiments. *P < 0.05 vs. vehicle alone.

Next, we determined the expression of cell-adhesion molecules at the protein level in HUVECs treated with estrogens by EIA. Eq treatment significantly increased the expression of E-selectin and ICAM-1 as compared with the levels observed in the control (*P* < 0.05; Fig 2A and 2B). Eq treatment also increased the protein expression of P-selectin and VCAM-1; however, these differences were not significant relative to control levels (Fig 2C and 2D). By contrast, treatment with other estrogens did not increase the protein levels of cell-adhesion molecules (Fig 2A–2D).

### Equilin, but not other estrogens, increases the number of monocytoid cells adhered to HUVECs

**Fig 3:**
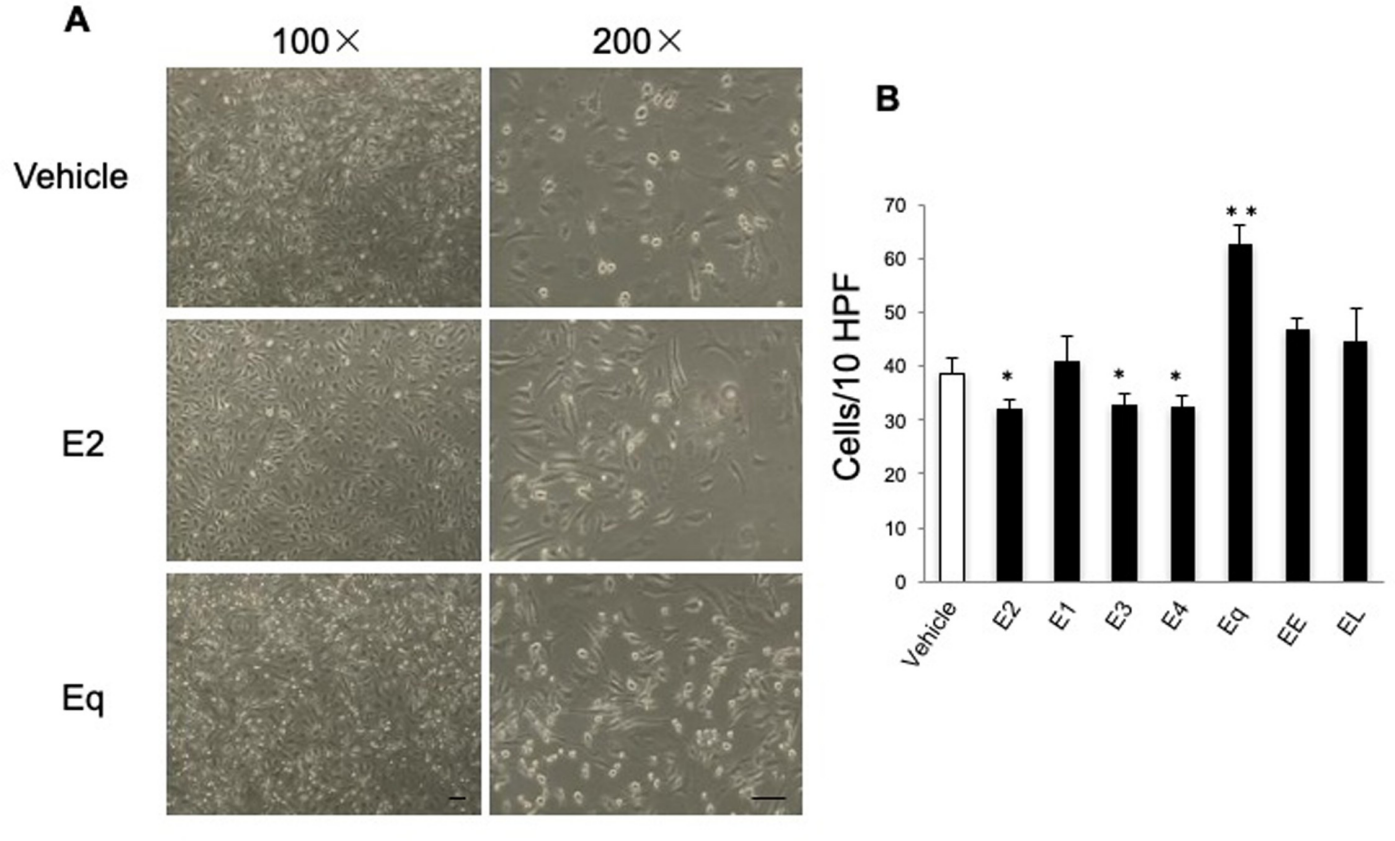
Effects of treatment with various estrogens on the adhesion of U937 monocytoid cells to HUVECs under flow conditions. HUVECs were treated with various estrogens as described in Fig 1. U937 monocytoid cells at 10,000 cells/mL were perfused over HUVEC monolayers, and adherent cells were counted 5 minutes after perfusion. (**A**) Representative micrographs of HUVECs in the flow-chamber system. The indicated small, round cells are adherent U937 monocytoid cells. Sample fields of cells at 40× magnification (*left*) and details of the cells (*right*) at high magnification (100×). Scale bar = 100 μm. (**B**) Total numbers of adherent cells in 10 randomly selected microscopic fields. Data are corrected by the blank and expressed as the mean ± SEM of six experiments. *P < 0.05 vs. vehicle alone. **P < 0.01 vs. vehicle alone.

We performed cell-adhesion assays to verify whether increased expression of cell-adhesion molecules promoted the adherence of U937 monocytoid cells to HUVECs. To mimic the physiological adherence of monocytes to endothelial cells under flow conditions, we used a flow-chamber system. During Eq exposure, the number of U937 monocytoid cells adhered to HUVECs significantly increased as compared with that observed in controls (*P* < 0.01; Fig 3A and 3B). By contrast, treatment with E2, E3, or E4 significantly reduced the number of monocytoid cells adhered to HUVECs (*P* < 0.05; Fig 3B).

### Is equilin action mediated by ERα and/or ERβ?

**Fig 4:**
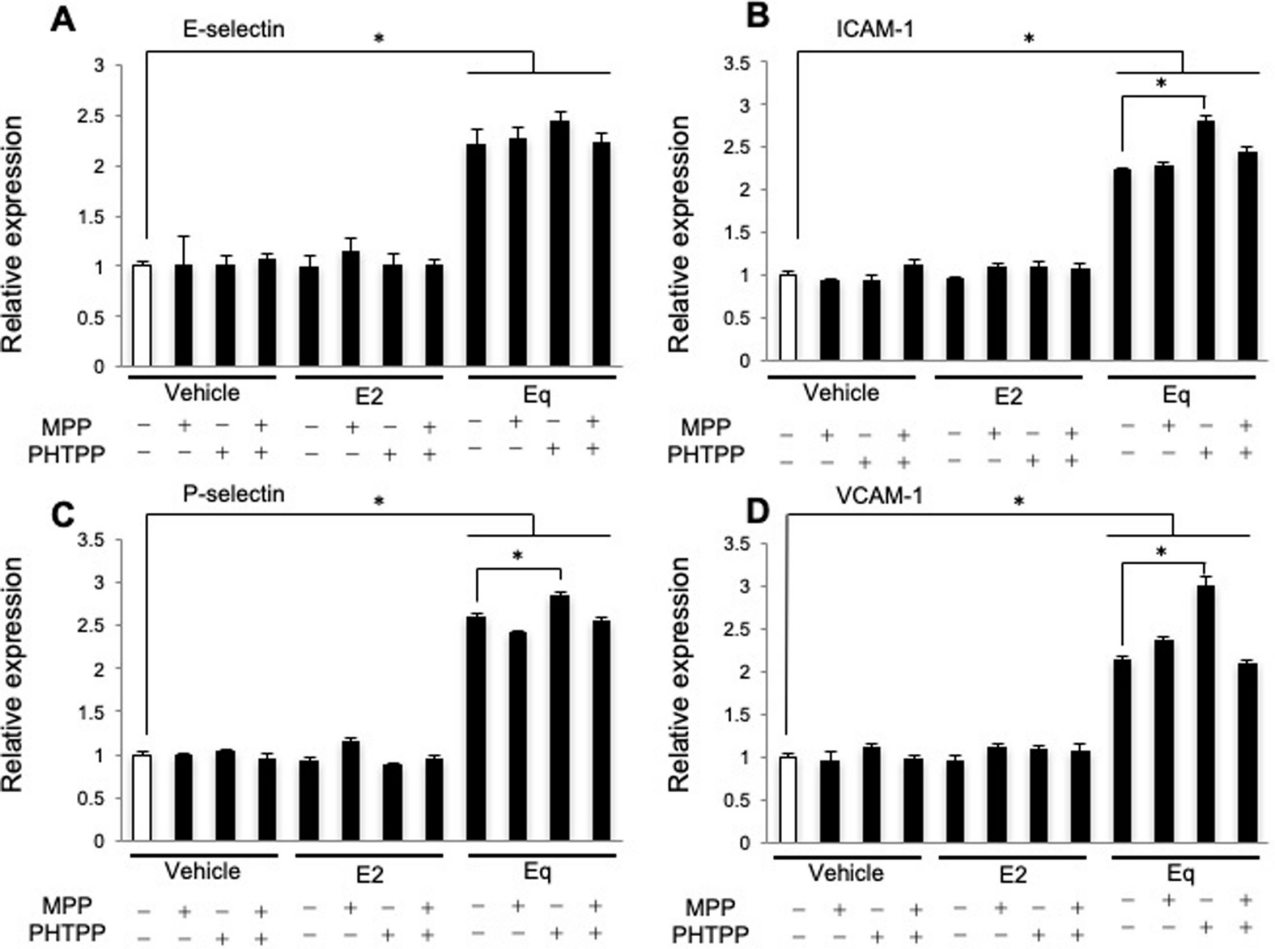
Effects of ERα and ERβ antagonists on the expression of endothelial-adhesion molecules during E2 or Eq treatment. HUVECs were treated with 1 nmol/L E2 or Eq in the presence or absence of 1 μmol/L MPP and/or 1 μmol/L PHTPP. Relative mRNA expression of genes encoding (**A**) E-selectin, (**B**) ICAM-1, (**C**) P-selectin, and (**D**) VCAM-1 in HUVECs was determined. Data are expressed as the mean ± SEM of three experiments involving assays performed in triplicate. *P < 0.05.

Estrogens exert many of their effects by binding to nuclear estrogen receptors, mainly ERα and ERβ. Therefore, we tested whether the elevated expression of adhesion molecules and the increased adherence of U937 cells to HUVECs during Eq exposure could be suppressed or enhanced by the ERα antagonist 1,3-bis (4-hydroxyphenyl)-4-methyl-5-[4-(2-piperidinylethoxy) phenol]-1H-pyrazole dihydrochloride (MPP) or the ERβ antagonist 4-[2-phenyl-5,7-bis(trifluoromethyl)pyrazolo[1,5-a]-pyrimidin-3-yl]phenol (PHTPP). Real-time PCR analysis revealed that PHTPP administration increased the expression of E-selectin during Eq exposure; however, the differences were not significant compared with control levels (Fig 4A). PHTPP administration resulted in moderate levels of Eq stimulation, resulting in increased ICAM-1, P-selectin, and VCAM-1 expression (*P* < 0.05; Fig 4B–4D). However, MPP administration did not affect Eq-induced changes in the expression of adhesion molecules (Fig 4A–4D). Interestingly, MPP + PHTPP administration also did not significantly alter the effect of Eq on the expression of adhesion molecules (Fig 4A–4D).

### Equilin enhances NF-κB activation by reducing ERβ expression

**Fig 5:**
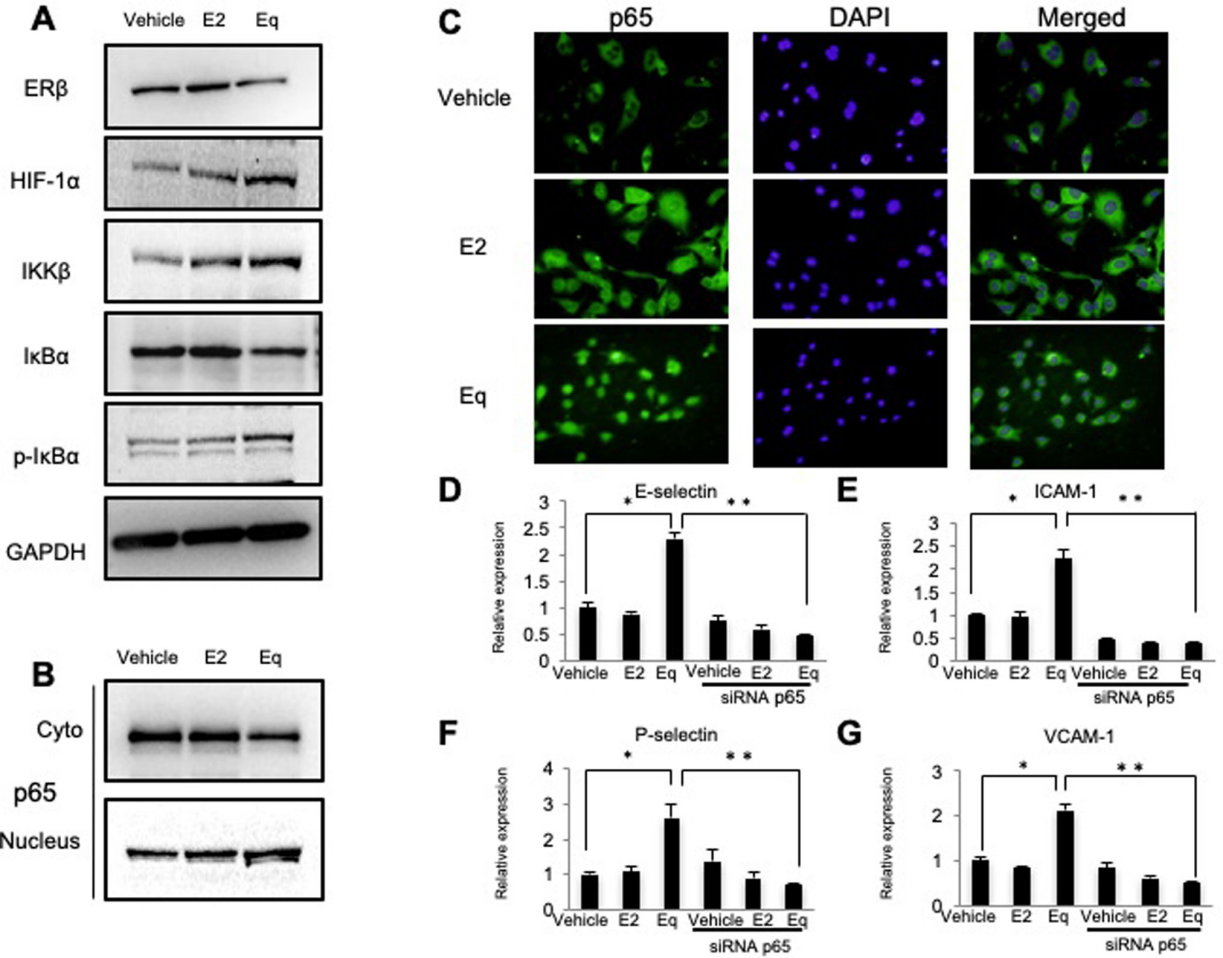
Effect of Eq treatment on the NF-κB-signaling pathway in HUVECs and differential expression of adhesion molecules in HUVECs following treatment with p65-specific or control siRNA. HUVECs were treated with 1 nmol/L E2 or Eq. (**A**) Effects of Eq on the expression of proteins involved in the NF-κB-signaling pathway (ERβ, HIF-1α, IKKβ, IκBα, and phosphorylated-IκBα) were evaluated by western blotting. (**B**) Concentrations of the p65 NF-κB subunit were measured in purified cytoplasmic or nuclear extracts by western blotting. (**C**) Representative images of immunofluorescence analysis of p65 localization in HUVECs at 200× magnification (*left*). The nuclear architecture is shown by 4′,6-diamidino-2-phenylindole staining (*middle*). All experiments were performed in triplicate with comparable results. Relative mRNA expression of genes encoding (**D**) E-selectin, (**E**) ICAM-1, (**F**) P-selectin, and (**G**) VCAM-1 in HUVECs transiently transfected for 24 or 48 hours with scrambled (control) or p65-specific siRNA and treated for 24 hours with or without 1 nmol/L E2 or Eq. Data are expressed as the mean ± SEM of three experiments involving assays performed in triplicate. *P < 0.05 vs. vehicle alone. **P < 0.01.

To understand the relationship between Eq stimulation and ERβ activity, we first examined the effect of Eq on ERβ expression. Western blot analysis revealed that Eq treatment reduced ERβ protein expression and increased the protein levels of HIF-1α, IKKβ, and phosphorylated-IκBα in HUVECs as compared with levels observed in the control. By contrast, we observed no significant change in E2-treated cells as compared with the control (Figs 5A and S2). Furthermore, western blot analysis showed that Eq treatment promoted p65 accumulation in HUVEC nuclear extracts as compared with levels observed in the control (Fig 5B). These findings indicate that Eq treatment promotes p65 nuclear translocation via the upregulation of the HIF-1α and IKKβ/IκBα canonical pathways. The actual translocation of p65 in HUVECs treated with Eq was evident based on immunofluorescence analysis (Fig 5C).

### Suppression of p65 prevents upregulation of adhesion molecules in HUVECs

To confirm that the Eq-induced upregulation of adhesion-molecule expression is mediated by the NF-κB-signaling pathway, we evaluated the effects of Eq treatment on HUVECs transfected with p65-specific siRNA, which is commonly used to specifically suppress the effect of p65. Real-time PCR analysis revealed that p65 suppression in HUVECs attenuated the Eq-induced upregulation of adhesion-molecule expression (*P* < 0.01; Fig 5D–5G). Additionally, we performed the experiments with the use of siRNA against ERβ. As expected, the down-regulation of ERβ with or without Eq treatment increased adhesion molecule expressions in HUVECs (S3 Fig).

## Discussion

To our knowledge, our results constitute the first demonstration that Eq, unlike other estrogens, increases the mRNA and protein expression of cell-adhesion molecules in HUVECs. Furthermore, we used a flow-chamber system to examine adherence between monocytoid cells and HUVECs. This system is capable of creating a laminar flow on the endothelial cell surface to impose shear stress and promote the rolling of monocytes on endothelial cells. Because the flow conditions more closely mimic physiological conditions than do rotated or static adhesion models [17–19], the assay provides a higher level of sensitivity for the assessment of monocyte–endothelial interactions than conventional cell-adhesion assays performed under static or agitated conditions [5,15,19]. Our results revealed that adherence between monocytoid cells and HUVECs under physiological conditions was enhanced by Eq stimulation *in vitro*. The adherence of monocytes to endothelial cells constitutes an important initial event in atherogenesis [13]. Monocytes adhered to endothelial cells translocate into the intima of vascular walls and promote the uptake of oxidative low-density lipoprotein cholesterol, and adhesion molecules, including E-selectin, P-selectin, ICAM-1, and VCAM-1, mediate the adherence between monocytes and endothelial cells [13]. While E-selectin causes rolling, ICAM-1 contributes to firm adhesion between monocytes and endothelial cells [14]. P-selectin and VCAM-1 are secretory, rather than membrane-bound, proteins that support the function of E-selectin and ICAM-1, respectively [14]. Furthermore, we previously reported that the knockdown of ICAM-1 leads to a reduction in monocyte adherence to HUVECs [5]. Therefore, the increased expression of adhesion molecules and the enhanced adhesion of monocytes to endothelial cells in postmenopausal women treated with ERT containing Eq may increase the risk of CHD. Conversely, our study showed that the administration of E2, E3, or E4 significantly decreased the adherence of monocytoid cells to HUVECs as compared with that observed in the control. These results suggest that natural estrogens, such as E2, E3, and E4, might exhibit protective effects against the risk of cardiovascular disease, which is consistent with results from a previous *in vitro* study [20].

Although the main component of CEE is estrone sulfate, this constitutes less than half of the compound. CEE contains at least 10 non-human estrogens divided into B-ring-saturated estrogens and B-ring-unsaturated estrogens, including Eq, which constitutes ~25% of CEE [9]. Bioassays have revealed that all 10 estrogens are biologically active [9,20]. The 2004 WHI trial suggested that CEE treatment alone did not reduce the risk of cardiovascular events in postmenopausal women [21]. Other randomized, controlled trials involving the daily administration of CEE as estrogen also failed to identify anti-atherosclerotic effects of estrogen [22]. However, there have been several *in vitro* studies suggesting that the natural estrogen E2 exerts beneficial effects on endothelial cells in terms of atherosclerosis risk [10–12]. A previous report showed that E2 strongly inhibits interleukin-1-mediated endothelial E-selectin and VCAM-1 induction and that this inhibition was abrogated by the administration of an ER antagonist [12]. E2 also induces arterial vasodilation by activating nitric oxide synthase via ERα in a non-genomic manner and inhibits the response to vascular injury, which leads to the development of atherosclerosis in a genomic manner [11]. There exists a degree of consensus regarding the atheroprotective effects of E2 on endothelial cells in postmenopausal women; however, basic studies investigating the effects of CEE-based estrogen (Eq or EL) on CHD risk in endothelial cells have not been reported.

ER-binding studies have indicated that Eq binds to both ERα and ERβ [9,23]. Therefore, to examine the mechanism associated with enhanced adhesion-molecule expression following Eq stimulation, we tested the effect of Eq on ERs through inhibitory experiments involving ERα and/or ERβ antagonists. Because our findings suggested that the Eq-mediated upregulation of adhesion-molecule expression might be associated with ERβ, we examined differences in ERβ expression in HUVECs treated with different estrogens. Based on the results, we subsequently focused on a novel mechanism involving the ERβ-related prevention of NF-κB activation via a reduction in HIF-1α levels [24]. Loss of ERβ stabilizes HIF-1α, and an inverse correlation between the expression of ERβ and HIF-1α/IKKβ in ERβ-knockout mice has been reported [24]. Additionally, HIF-1α promotes NF-κB expression and transcriptional activation via the IKKβ/IκBα canonical pathway [25–27]. The inactivation of the NF-κB pathway results in the cytoplasmic localization of the heterodimeric complex involving p65 and p50. When activated by external stimuli, such as cytokines, lipopolysaccharides, or viruses, IκB kinases, including IKKβ, phosphorylate IκBα, resulting in the translocation of the p65-p50 complex to the nucleus to regulate target-gene transcription [28]. Moreover, all adhesion molecules tested in this study are known target genes of NF-κB, as well as inflammatory cytokines [29–32]. Therefore, our results suggest that increased adhesion-molecule expression and enhanced adhesion of U937 cells to HUVECs under Eq stimulation are induced in part by NF-κB activation following the decreased expression of ERβ. Conversely, we confirmed that the suppression of ERβ resulted in the upregulation of adhesion molecule expressions in HUVECs. Furthermore, our results are consistent with those of previous studies reporting that CEE administered as ERT to postmenopausal women increases the levels of serum C-reactive protein (CRP), a target gene of NF-κB, whereas transdermal E2 administration has no effect on CRP levels [33–35].

Recent clinical trials have showed that pure natural estrogen (E2) prevents atherosclerosis [36,37] and that ERT with E2 immediately following menopause significantly reduces the risk of mortality, heart failure, and myocardial infarction in postmenopausal women [36]. Another clinical trial suggested that oral intake of E2 is associated with reduced progression of non-symptomatic subclinical atherosclerosis in postmenopausal women when therapy is initiated within six years following menopause [37]. Several clinical studies comparing CEE with E2 have shown that CEE treatment upregulates levels of certain inflammatory cytokines and triglycerides as compared with levels resulting from treatment with transdermal E2 [6,7]. Additionally, CEE use, as compared with oral E2 administration, is associated with a higher risk of venous thrombosis and possibly myocardial infarction [6,7]. Similar to our results, the findings of these clinical trials suggest that oral or transdermal E2 intake may be more favorable than CEE administration for ERT in postmenopausal women in terms of atherosclerosis risk.

There are some limitations of our *in vitro* study. CEE is usually administered for ERT at a daily dose of 0.625 mg, and Eq constitutes ~25% of CEE. Therefore, considering clinical use, we used all estrogens except EE, at a concentration of 1 nmol/L. Our preliminary experiments revealed a dose-dependent effect of Eq at concentrations of 100 pmol/L to 10 nmol/L on adhesion-molecule expression (data not shown). However, the actual effects of CEE on endothelial cells are attributable to a sum of the effects associated with other estrogens, with final concentrations defined following the first pass through the liver. *In vivo* experiments will be needed to verify whether long-term exposures of Eq actually increase atherosclerosis risk.

In conclusion, Eq increases adhesion-molecule expression on HUVECs and enhances the adherence of monocytoid cells to HUVECs *in vitro*. Our findings also indicate that Eq-induced effects on endothelial cells are mediated by a reduction in ERβ expression, resulting in NF-κB activation. These findings suggest that E2 may be preferable for ERT in terms of the adhesion of monocytes at the onset of atherosclerosis. Further studies comparing Eq with E2 are needed to investigate differential impacts on atherosclerosis.

## Supporting information

S1 Fig Time dependency related to the effect of Eq on adhesion molecule expression in HUVECs. Steroid-deprived and serum-starved HUVECs were treated for 6 to 24 h in the presence of Eq. The relative mRNA expression of genes encoding (**A**) E-selectin, (**B**) ICAM-1, (**C**) P-selectin, and (**D**) VCAM-1 was measured by real-time PCR. Data are expressed as the mean ± SEM of three experiments involving assays performed in triplicate. *P < 0.05 vs. vehicle alone.
(TIFF)

S2 Fig Quantification of the relative levels of expressions in Fig 5A. Densitometric analysis of blots in Fig 5A was performed. Relative protein expression of genes encoding (**A**) ERβ, (**B**) HIF-1α, (**C**) IKKβ, (**D**) IκBα, and (**E**) phosphorylated-IκBα was determined. Data are expressed as the mean ± SEM of three experiments involving assays. *P < 0.05 vs. vehicle alone.
(TIFF)

S3 Fig Relative expression of adhesion molecules in HUVECs following treatment with ERβ-specific or control siRNA. Relative mRNA expression of genes encoding (**A**) ERβ, (B) E-selectin, and (**C**) ICAM-1 in HUVECs transiently transfected for 48 hours with scrambled (control) or p65-specific siRNA and treated for 24 hours with or without 1 nmol/L E2 or Eq. Data are expressed as the mean ± SEM of three experiments involving assays performed in triplicate. *P < 0.05 vs. vehicle alone.
(TIFF)

S1 Table Summary of oligonucleotide primers used for RT-PCR. (TIFF)
