# Supplementary figures and images for: Equilin in conjugated equine estrogen increases monocyte-endothelial adhesion via NF-κB signaling
Source: PLoS One. 2019 Jan 30;14(1):e0211462. doi: 10.1371/journal.pone.0211462 (PMC6353580; doi:10.1371/journal.pone.0211462)

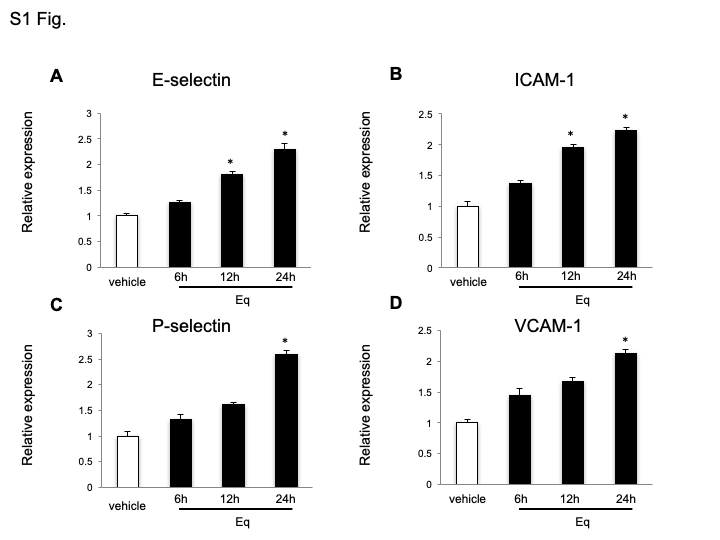

Supplement: S1 Fig — Steroid-deprived and serum-starved HUVECs were treated for 6 to 24 h in the presence of Eq. The relative mRNA expression of genes encoding (A) E-selectin, (B) ICAM-1, (C) P-selectin, and (D) VCAM-1 was measured by real-time PCR. Data are expressed as the mean ± SEM of three experiments involving assays performed in triplicate. *P < 0.05 vs. vehicle alone. (TIFF) [file pone.0211462.s001.tiff]

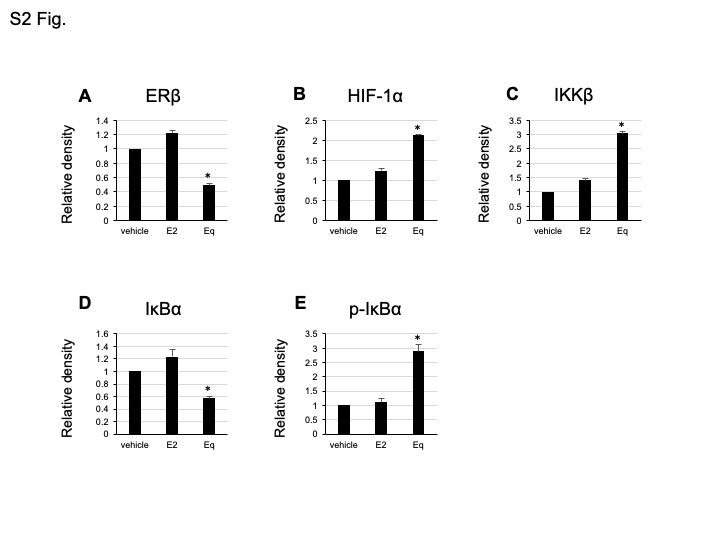

Supplement: S2 Fig — Densitometric analysis of blots in Fig 5A was performed. Relative protein expression of genes encoding (A) ERβ, (B) HIF-1α, (C) IKKβ, (D) IκBα, and (E) phosphorylated-IκBα was determined. Data are expressed as the mean ± SEM of three experiments involving assays. *P < 0.05 vs. vehicle alone. (TIFF) [file pone.0211462.s002.tiff]

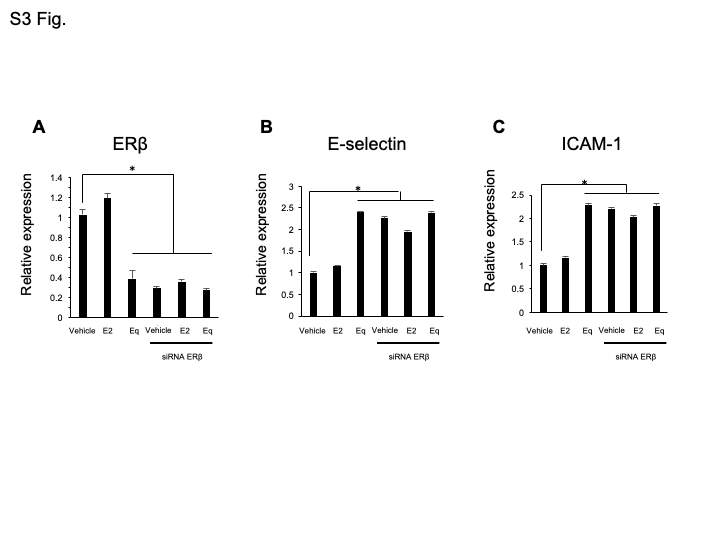

Supplement: S3 Fig — Relative mRNA expression of genes encoding (A) ERβ, (B) E-selectin, and (C) ICAM-1 in HUVECs transiently transfected for 48 hours with scrambled (control) or p65-specific siRNA and treated for 24 hours with or without 1 nmol/L E2 or Eq. Data are expressed as the mean ± SEM of three experiments involving assays performed in triplicate. *P < 0.05 vs. vehicle alone. (TIFF) [file pone.0211462.s003.tiff]

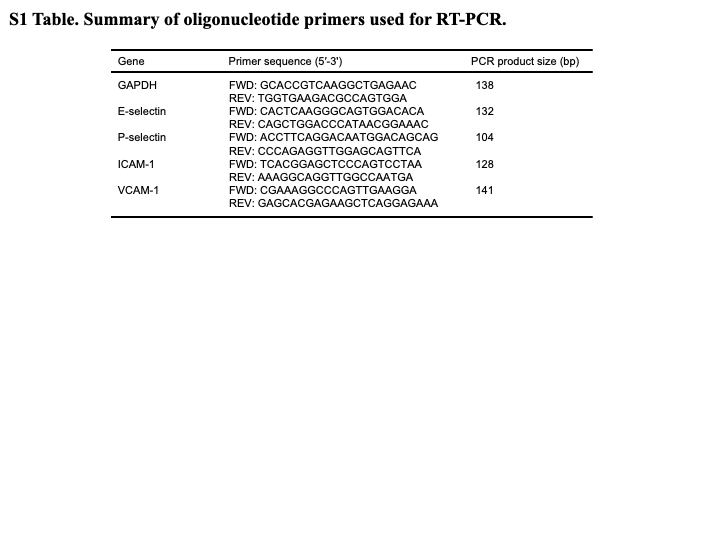

Supplement: S1 Table — (TIFF) [file pone.0211462.s004.tiff]
